# A high-resolution climate simulation dataset for the past 540 million years

**DOI:** 10.1038/s41597-022-01490-4

**Published:** 2022-06-28

**Authors:** Xiang Li, Yongyun Hu, Jiaqi Guo, Jiawenjing Lan, Qifan Lin, Xiujuan Bao, Shuai Yuan, Mengyu Wei, Zhibo Li, Kai Man, Zihan Yin, Jing Han, Jian Zhang, Chenguang Zhu, Zhouqiao Zhao, Yonggang Liu, Jun Yang, Ji Nie

**Affiliations:** 1 Laboratory for Climate and Ocean-Atmosphere Studies, Department of Atmospheric and Oceanic Sciences, School of Physics, Peking University, Beijing, China; 2 Department of Atmospheric Science, School of Environmental Studies, China University of Geosciences, Wuhan, China

**Keywords:** Palaeoclimate, Climate and Earth system modelling, Atmospheric science

## Abstract

The Phanerozoic Eon has witnessed considerable changes in the climate system as well as abundant animals and plant life. Therefore, the evolution of the climate system in this Eon is worthy of extensive research. Only by studying climate changes in the past can we understand the driving mechanisms for climate changes in the future and make reliable climate projections. Apart from observational paleoclimate proxy datasets, climate simulations provide an alternative approach to investigate past climate conditions of the Earth, especially for long time span in the deep past. Here we perform 55 snapshot simulations for the past 540 million years, with a 10-million-year interval, using the Community Earth System Model version 1.2.2 (CESM1.2.2). The climate simulation dataset includes global distributions of monthly surface temperatures and precipitation, with a 1° horizontal resolution of 0.9° × 1.25° in latitude and longitude. This open access climate dataset is useful for multidisciplinary research, such as paleoclimate, geology, geochemistry, and paleontology.

## Abstract (summary)

**Table:** 

Measurement(s)	climate change • temperature • volume of hydrological precipitation
Technology Type(s)	computational modeling technique
Factor Type(s)	surface temperature • precipitation
Sample Characteristic - Organism	monthly surface temperatures and precipitation
Sample Characteristic - Environment	climate system
Sample Characteristic - Location	global

## Background & Summary

The Phanerozoic Eon, comprising the Paleozoic, Mesozoic, and Cenozoic Eras, covers the last 542 million years (Myr) of Earth’s history, which is about 12% of the history of our planet^1^. Climate states over the Phanerozoic Eon consist of alternating warm and cool intervals. The classical feature of Phanerozoic climate history is the “double hump” temperature variations^2–4^, with warm climate in the Early Paleozoic, cooler climate in the Late Paleozoic, followed by warmer climate in the Mesozoic and Early Cenozoic and cooler climate in the Late Cenozoic^5^.

It is acknowledged that proxy records provide precious evidence for paleoclimate studies. However, due to unavoidable uncertainties of proxy records, sparse records with limited spatial coverage, and the fact that many proxies may respond to multiple climatic variables or even non-linear combinations of variables^6^, it is far from adequate for proxy records to provide global climate patterns. For example, only climatic zonation has been inferred from compilations of lithologic climate indicators, such as coals and evaporites in the Phanerozoic Eon^5,7–10^. Alternatively, climate models are a useful tool to simulate paleoclimates. Especially, climate models are able to generate global distributions of climate variables with rather fine spatial resolution, and climate variables are self-constrained by dynamical, physical and chemical processes in climate models. It not only makes up the defects of proxy records but also can be used to check the reliability of proxies.

Paleoclimate simulations for a long span of time are computationally expensive and time-consuming. To our knowledge, there have been few simulation studies covering the whole Phanerozoic Eon. Landwehrs *et al*.^11^ performed 40 time-slice simulations for the period from 255 million years ago (Ma) to 60 Ma, using the CLIMBER-3α Earth System Model of Intermediate Complexity (EMIC) that has a relatively coarse spatial resolution. The Bristol Research Initiative for the Dynamic Global Environment (BRIDGE) group at University of Bristol has produced large datasets of paleoclimate simulations^12–14^. Especially, Valdes *et al*.^15^ conducted 109 time-slice simulations that cover the entire Phanerozoic, using a coupled atmosphere–ocean–vegetation model.

Here, we perform 55 snapshot simulations for the Phanerozoic Eon with a time interval of 10 Myr, using the Community Earth System Model version 1.2.2 (CESM1.2.2). The dataset has a high resolution of 0.9° × 1.25° in latitude and longitude. It offers elaborate global distributions of monthly surface temperatures and precipitation throughout the Phanerozoic Eon. It can be referenced and cross validated by research fields across geology, paleobiology, geochemistry, etc.

## Methods

### CESM1.2.2

The CESM1.2.2 is a coupled climate model that consists of atmosphere, ocean, land, sea-ice and river components, which are linked through a coupler that interacts and exchanges state information and fluxes among the components^16^. The fully coupled CESM has been successfully implemented for simulating past and modern climates^17–24^.

Two versions of the CESM1.2.2 are used in this study. One is a fully-coupled version which uses a T31 spectral dynamical core for the atmospheric (Community Atmosphere Model version 4, CAM4^25^) and land (Community Land Model version 4, CLM4^26^) components (horizontal grid of 3.75° × 3.75°) with 26 atmospheric layers in the vertical. The ocean (Parallel Ocean Program version 2, POP2^27^) and sea-ice (Community Ice CodE version 4, CICE4^28^) components employ a nominal 3° irregular horizontal grid (referred to as g37) with 60 oceanic layers in the vertical. The River Transport Model (RTM) has a default resolution of 0.5° × 0.5° in latitude and longitude, which directs all runoff to oceans, without interior drainage loops based on computations of surface topography.

The other one is the atmosphere-land-coupled version which applies the finite-volume dynamical core with a 1° atmosphere (f09: 0.9° × 1.25° latitude versus longitude) with the same vertical levels as T31. For this version of simulations, the model is driven by prescribed climatological monthly mean sea surface temperatures (SSTs), sea-ice (SI), and annual mean land vegetation, which are derived from the T31_g37 equilibrium simulations. Model performance of these two versions has been assessed and validated for modern conditions^29,30^.

CLM4 incorporates a carbon–nitrogen (CN) cycle component that is prognostic in carbon, nitrogen and vegetation phenology^31^. Note that here carbon and nitrogen fluxes are purely diagnostic and are not passed to the atmosphere, and thus do not influence atmospheric CO_2_ concentrations^26^. Even though the carbon fluxes are only diagnostic, the CN model will have an influence on the climate simulation because seasonal and interannual vegetation phenology, i.e., leaf area index (LAI) and vegetation height, is prognostic^30^. In addition, CLM4 has the option to run the CN model with dynamic vegetation (CNDV)^32,33^. CNDV modifies the CN framework to implement plant biogeography updates, and simulates unmanaged vegetation including tree, grass, and also shrub^34^ plant functional types (PFTs). It is worth pointing out that the PFTs are the same for all simulations, and that plant evolution is not considered in this study. Establishment of new PFTs is based on the warmest minimum monthly air temperature and minimum annual growing degree-days above 5 °C, and minimum precipitation of 100 mm yr^−1^ is required to introduce new PFTs. Survival is based on the coldest minimum monthly air temperature^35^. PFTs must be able to survive in order to establish. CNDV simulates a reasonable present-day distribution of PFTs but underestimates tundra vegetation cover^33^. Here the CNDV is active only in the fully-coupled T31_g37 model to generate PFTs.

### Experimental set-up

#### Boundary conditions

We perform 54 time-slice simulations from 540 Ma to 10 Ma, with a time interval of 10 Myr between each two snapshot simulations. The pre-industrial simulations will be described later. Paleogeographic maps from the paleo-digital elevation model (paleoDEM)^36^ are used here as boundary conditions. The paleoDEM elaborates the changing distribution of deep oceans, shallow seas, lowlands, and mountainous regions, which is an estimate of the elevation of the land surface and depth of the ocean basins measured in meters with a resolution of 1° × 1°. The digital paleogeographic maps are interpolated according to model resolutions with minor changes of the land-sea masks for the purpose of model stability. Note that the paleogeographic maps do not include information of ice sheets, and that there are no prescribed ice sheets for simulations from 540 Ma to 10 Ma. The initial land surface is set as warm grassland, and the surface soil is set to a uniform loam.

#### CO_2_ concentrations and solar radiation

Different from previous simulation studies, here we use reconstructed global mean surface temperatures (GMSTs; All GMSTs herein are annual means.)^37,38^ to constrain our simulations, rather than using reconstructed CO_2_ concentrations. This alternative approach of simulations is equivalent to using reconstructed GMSTs to “predict” atmospheric CO_2_ concentrations. Thus, it is worthy here to briefly introduce the methodology of the GMST reconstruction^37,38^.

The time series of Phanerozoic GMSTs was reconstructed by combining estimations of pole-to-equator temperature gradients derived from lithologic records and tropical temperatures derived from oxygen isotopes^5^. First, five major Köppen belts are mapped, using lithologic indicators of climate (tillites, evaporites, coals, bauxites, etc.). Based on modern climate conditions, temperatures are assigned to each of the Köppen belts, so that the zonal mean pole-to-equator temperature profiles can be obtained. Second, oxygen isotopic values are converted to estimate tropical temperatures, with modifications based on geological and paleontological considerations. As a result, GMSTs can be calculated using meridional temperature profiles and tropical temperatures. Readers can refer to Scotese *et al*.^5^ for comprehensive description of the methodology in deriving the GMSTs and its uncertainties.

For simulations from 540 Ma to 10 Ma, CO_2_ concentrations are tuned until simulated GMSTs are asymptotic to reconstructed GMSTs within ± 0.5 °C. In the process of tuning CO_2_ concentrations, we first estimate the required CO_2_ concentration according to the climate sensitivity of the T31_g37 version and use it to force the model. After running the model for about 2000 years, we check the simulated GMST and decide to increase or decrease the CO_2_ concentration. We need to try a few times until the difference between the simulated GMST and the reconstruction value is within ± 0.5 °C at the equilibrium state in which the net radiation at the top of the atmosphere (TOA), averaged over the last 100 model years, is within ± 0.1 W m^−2^. Except for the CO_2_ concentration, all other atmospheric compositions are set to the pre-industrial (PI) values.

Solar radiation is linearly increased from 1302 W m^−2^ at 540 Ma to 1361 W m^−2^ at the present, with an increasing rate of about 0.08% per 10 Myr^39^. Orbital parameters are set to the present values. A summary of CO_2_ concentrations and solar radiation used in our simulations is given in Table 1.

**Table 1:** Atmospheric CO_2_ concentrations, solar radiation, and simulated global and annual mean surface temperatures and precipitation for the 55 snapshot simulations in this study.

Simulation	Year (Ma)	CO_2_ (×280 ppmv)	Solar radiation (W m^−2^)	Surface temperature (°C)	Precipitation (mm yr^−1^)
1	540	28	1302.20	26.4	1301
2	530	27	1303.29	26.3	1311
3	520	23	1304.38	26.0	1324
4	510	23	1305.47	25.9	1316
5	500	25	1306.56	26.0	1329
6	490	25	1307.65	26.3	1346
7	480	24	1308.74	26.7	1366
8	470	23	1309.83	26.5	1358
9	460	16	1310.92	24.1	1286
10	450	6	1312.00	19.2	1177
11	440	6	1313.09	18.4	1144
12	430	7	1314.18	21.2	1233
13	420	6	1315.27	21.3	1252
14	410	8	1316.36	21.8	1238
15	400	9	1317.45	23.0	1258
16	390	18	1318.54	25.1	1334
17	380	15	1319.63	25.1	1329
18	370	11	1320.71	22.9	1270
19	360	8	1321.80	20.8	1202
20	350	8	1322.89	19.8	1187
21	340	8	1323.98	20.2	1200
22	330	7	1325.07	19.2	1159
23	320	6	1326.16	17.5	1102
24	310	2.8	1327.25	13.5	1014
25	300	3.5	1328.34	15.8	1070
26	290	2.3	1329.42	12.5	970
27	280	3	1330.51	15.2	1022
28	270	6.5	1331.60	19.2	1090
29	260	10	1332.69	22.0	1152
30	250	25	1333.78	25.8	1195
31	240	28	1334.87	25.5	1162
32	230	24	1335.96	25.1	1162
33	220	20	1337.05	24.7	1179
34	210	20	1338.14	24.2	1152
35	200	17	1339.22	22.9	1101
36	190	10	1340.31	20.3	1110
37	180	10	1341.40	19.8	1087
38	170	7	1342.49	19.1	1138
39	160	6	1343.58	19.4	1127
40	150	7	1344.67	19.8	1127
41	140	10	1345.76	20.6	1135
42	130	9	1346.85	21.3	1176
43	120	9	1347.93	22.3	1212
44	110	9	1349.02	23.0	1235
45	100	9	1350.11	23.8	1259
46	90	8	1351.20	24.2	1276
47	80	7	1352.29	24.3	1284
48	70	8	1353.38	23.4	1208
49	60	6.5	1354.47	22.3	1194
50	50	7	1355.56	22.6	1211
51	40	4.9	1356.64	20.2	1148
52	30	2.6	1357.73	16.1	1044
53	20	2.6	1358.82	15.6	1037
54	10	2.6	1359.91	15.6	1033
55	Pre-industrial	1	1360.89	14.3	1048

#### Two-step simulations

For the first step of simulations, the fully coupled T31_g37 CESM1.2.2 is used. The key in this step of simulations is to tune CO_2_ concentrations until the simulated GMSTs are close to reconstructions at equilibrium states, that is, GMST differences between simulations and reconstructions are within ± 0.5 °C. We initialize surface temperatures of the atmospheric component model with zonally uniformly distributed values ranging from 20 °C at the equator to 1 °C at the poles for all simulations. Ocean temperature is initialized with a globally uniform vertical profile. Three types of vertical temperature profiles are chosen. For cold periods, the vertical temperature profile varies from 15 °C at the surface to 2 °C at the bottom. For warm periods, the vertical profile varies from 20 °C to 4 °C. For hot periods, the vertical profile varies from 24 °C to 8 °C. The reason why we choose the three types of vertical temperature profiles is to have simulations reach equilibrium states faster. The initial ocean salinity is globally and vertically uniform, with a value of 35 psu, for all simulations. SI and PFTs are initially set to zero. In all the simulations, there are no prescribed ice sheets, except for the PI simulation.

All simulations are integrated for more than 4000 model years to reach equilibrium states at which the net radiation at the TOA, averaged over the last 100 model years, is less than 0.1 W m^−2^. Some of the simulations are even run for more than 6000 model years. The model was run with the CNDV model to generate global vegetation cover.

For the second step of simulations, repeating annual cycles of monthly SSTs and SI, as well as annual mean vegetation cover, averaged over the last 100 model years in the first step of simulations are used to drive the f09 atmosphere-land-coupled model. Paleogeography, CO_2_ concentrations, and solar radiation remain the same as those in the first step. All simulations are integrated for 100 model years so that the atmosphere model reaches equilibrium states. The results presented here are the averages over the last 60 model years.

#### Pre-industrial simulation

For reference, the PI simulations are performed with the modern continental configuration and the model default PI vegetation cover and ice sheets. CO_2_ concentration is set to the PI value, i.e., 280 ppmv. The solar constant is set as 1361 W m^−2^. All other conditions are set to the PI default values. Note that the PI f09 simulation is not driven by the SSTs, SI, and vegetation derived from the T31_g37 run, but by the model default PI conditions.

## Data Records

The datasets are constructed in the form of the NetCDF File ‘High_Resolution_Climate_Simulation_Dataset_540_Myr.nc’ and can be found in the Figshare repository^40^. Climate variables include monthly surface temperatures (T; unit: °C; Not surface air temperatures), precipitation (P; unit: mm month^−1^), fraction of surface land area (LANDFRAC; unit: fraction), surface geopotential (PHIS; unit: m^2^ s^−2^), surface albedo (SALB; unit: fraction), and zonal (U; unit: m s^−1^) and meridional (V; unit: m s^−1^) winds at 1000 hPa, averaged over the last 60 model years. T, P, SALB, U, and V have the dimensions of 55 (simulation) × 12 (month) × 192 (latitude) × 288 (longitude). LANDFRAC and PHIS have the dimensions of 55 (simulation) × 192 (latitude) × 288 (longitude).

**Fig. 1:**
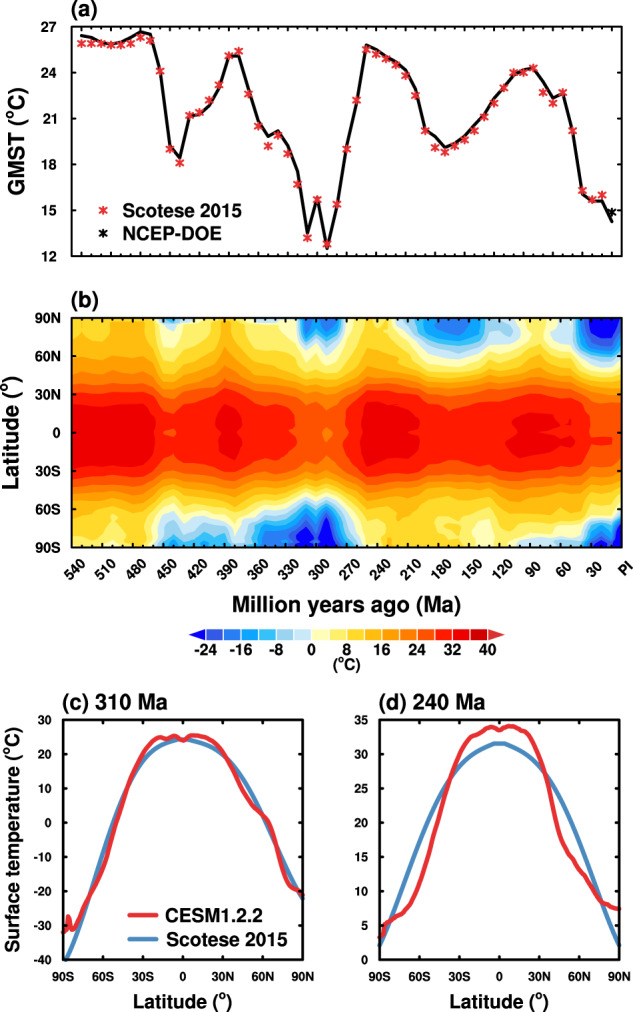
(**a**) Time series of annual mean GMSTs for the past 540 million years. Black line denotes simulated annual mean GMSTs. The red asterisks denote reconstructed GMSTs by Scotese^37,38^. The black asterisk denotes the annual mean GMST averaged for 1979–2020, using the data from NCEP-DOE Reanalysis 2^43^. **(b)** Variations of simulated annual and zonal mean surface temperatures for the past 540 million years. Annual and zonal mean surface temperature profiles for **(c)** 310 Ma and **(d)** 240 Ma. Red line denotes simulated surface temperatures using CESM1.2.2. Blue line denotes reconstructed surface temperatures by Scotese^37,38^. GMST, global mean surface temperature; NCEP-DOE, National Center for Environmental Prediction-Department of Energy; Ma, million years ago; CESM1.2.2, Community Earth System Model version 1.2.2.

Figure 1a shows the time series of simulated GMSTs (black line), which range from about 12 °C to about 27 °C over the past 540 Myr. The simulated GMSTs match the reconstructed GMSTs by Scotese^37,38^ (red asterisks) very well. A full list of simulated annual mean GMST values is presented in Table 1. Figure 1b shows the evolution of simulated zonal mean surface temperatures. First, zonal mean surface temperatures also demonstrate the “double hump” feature. Second, zonal mean surface temperatures show weaker meridional gradients during warmer periods such as the Early Paleozoic and the Mesozoic, and sharper meridional gradients during cooler periods such as the Late Paleozoic and the Late Cenozoic.

It is notable that the simulated equator-to-pole profiles of zonal mean surface temperatures are different from reconstructions by Scotese^37,38^, although the simulated and reconstructed GMSTs are almost the same. For example, Fig. 1c,d show zonal mean surface temperature profiles of cold climate (310 Ma) and hot climate (240 Ma), respectively. In both plots, the simulated surface temperatures are higher than reconstructions in the tropics and lower at middle latitudes, with sharper meridional gradients in the subtropics.

**Fig. 2:**
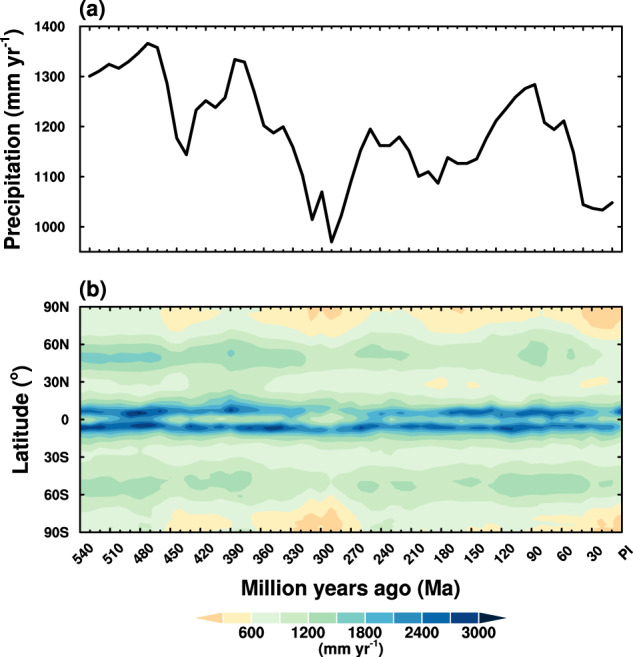
Simulated precipitation for the past 540 million years. **(a)** Time series of global and annual mean precipitation, and **(b)** variations of simulated annual and zonal mean precipitation.

Figure 2a shows the time series of simulated global and annual mean precipitation. It ranges between 950 mm yr^−1^ and 1400 mm yr^−1^ over the past 540 Myr. A full list of simulated global and annual mean precipitation is shown in Table 1. Annual and zonal mean precipitation is shown in Fig. 2b. There are two rain bands near the equator, with the maximum precipitation of about 3000 mm yr^−1^. Note that the double rain bands could be due to the “double ITCZ” bias, which is a common problem for coupled atmosphere-ocean climate models^41,42^. The secondary rain bands are around 50°N and S, with the largest precipitation of about 1600 mm yr^−1^. Two relatively dry bands are around 30 °N and S, which are the subtropical dry zones. Precipitation in both polar regions is the lowest.

**Fig. 3:**
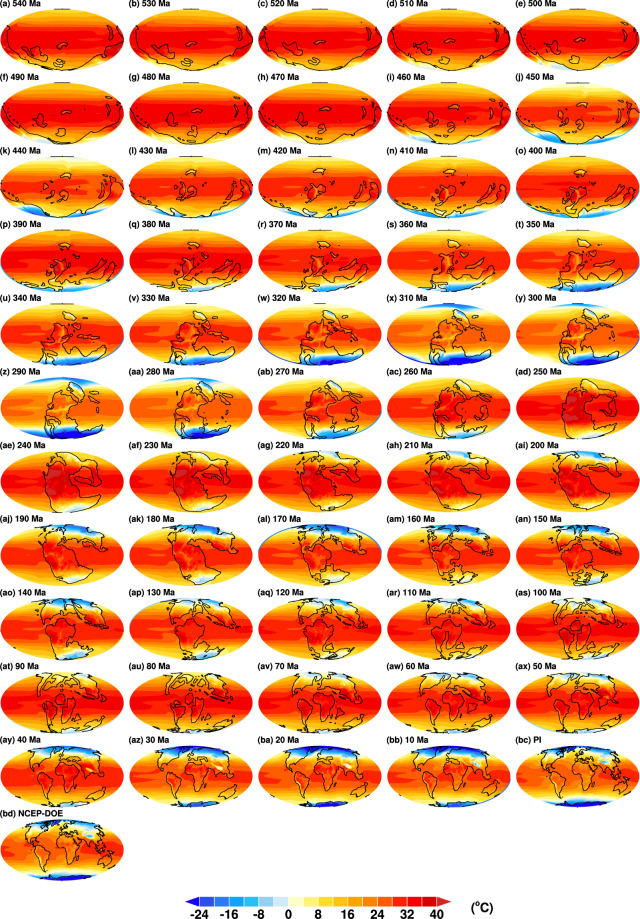
Global distributions of simulated annual mean surface temperatures from 540 Ma to the pre-industrial **(a**–**bc)**. Panel **(bd)** is the annual mean surface temperature averaged over 1979–2020, using the data from NCEP-DOE Reanalysis 2^43^. Ma, million years ago; NCEP-DOE, National Center for Environmental Prediction-Department of Energy.

**Fig. 4:**
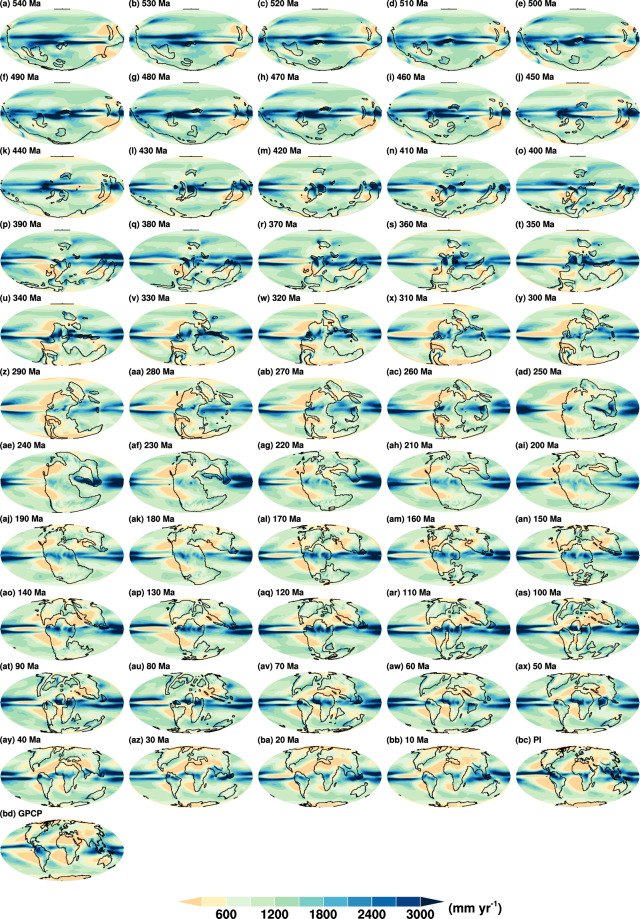
Global distributions of simulated annual mean precipitation from 540 Ma to the pre-industrial **(a**–**bc)**. Panel **(bd)** is the annual mean precipitation averaged over 1979–2020, using the data from GPCP version 2.3^44^. Ma, million years ago; GPCP, Global Precipitation Climatology Project.

Figures 3 and 4 demonstrate global maps of annual mean surface temperatures and precipitation of the 55 snapshot simulations, respectively. Figures 3bd and 4bd show modern surface temperatures and precipitation averaged over 1979–2020, respectively. The temperature and precipitation datasets are reanalysis from the National Center for Environmental Prediction-Department of Energy (NCEP-DOE) Reanalysis 2^43^ and the Global Precipitation Climatology Project (GPCP) Climate Data Record (CDR) version 2.3^44^, respectively. Figure 3w–aa show that annual mean surface temperatures over the south polar continents are as low as −24 °C, indicating formation of glaciers over 320–280 Ma. Similarly, Figs. 3az–bc show that annual mean surface temperatures over both polar regions are below −20 °C. It suggests that polar ice caps could start from 30 Ma.

## Technical Validation

**Fig. 5:**
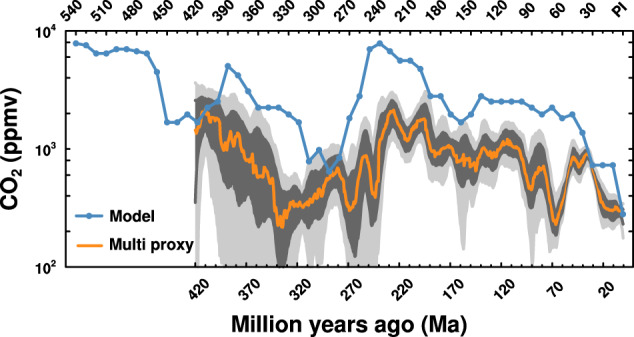
Time series of atmospheric CO_2_ concentrations. The blue line shows CO_2_ concentrations used in our simulations, and the orange line is the most likely LOESS fit of multi-proxy CO_2_ concentrations compiled from the literature (as Fig. 1 in Foster *et al*.^45^). 68% and 95% confidence intervals of the LOESS fit are shown as dark and light grey shadings. LOESS, locally estimated scatterplot smoothing.

As mentioned in the Methods section, atmospheric CO_2_ concentrations are predicted by reconstructed GMSTs in the present study. Figure 5 compares CO_2_ concentrations between our simulations (blue line) and reconstructions (orange line and shadings)^45^. Clearly, CO_2_ concentrations in our simulations are several times higher than reconstructions. This is due to two major reasons. One is the equilibrium climate sensitivity (ECS) of CESM1.2.2, and the other one is related to the dynamic vegetation model used in our simulations.

### Equilibrium climate sensitivity of CESM1.2.2

The ECS of the T31_g37 CESM1.2.2 used here is 2.9 °C^46^. According to the Sixth Assessment Report of the Intergovernmental Panel on Climate Change (IPCC-AR6)^47^, the likely range of ECS is between 2.5 °C and 4.0 °C, and the best estimate value is 3.0 °C. Thus, the ECS of T31_g37 CESM1.2.2 is close to the best estimate in IPCC-AR6.

### Uncertainty from the dynamic vegetation model

It is found that the dynamic vegetation model used here generates rather low areal vegetation coverage in all simulations, which could cause cold biases in the simulated GMSTs. To verify this, we perform two PI simulations, one with the CNDV, and the other one with prescribed default vegetation (64% vegetation cover). The former generates 25% vegetation cover per land grid cell on average, and the corresponding GMST is 10.6 °C. The latter yields a GMST of 13.1 °C, 2.5 °C higher than that with the CNDV. It suggests that the CNDV indeed causes cold biases and leads to an overestimation of the CO_2_ concentrations by about 1.8 times as the ECS of 2.9 °C per doubling atmospheric CO_2_ is considered.

## Data Availability

The source code of CESM1.2.2 can be accessed at https://www.cesm.ucar.edu/models/cesm1.2. The scripts used to generate the datasets and figures have been written using the NCAR Command Language version 6.6.2 (NCL6.6.2)^48^ and are available in the Figshare repository^40^.
